# Prophylactic Dermatologic Treatment of Afatinib-Induced Skin Toxicities in Patients with Metastatic Lung Cancer: A Pilot Study

**DOI:** 10.1155/2019/9136249

**Published:** 2019-01-31

**Authors:** Maria Pia Fuggetta, Maria Rita Migliorino, Serena Ricciardi, Giorgia Osman, Daniela Iacono, Alvaro Leone, Alessandra Lombardi, Giampietro Ravagnan, Stefania Greco, Daniele Remotti, Maria Concetta Pucci Romano

**Affiliations:** ^1^Institute of Translational Pharmacology, CNR, Rome, Italy; ^2^UOSD of Oncologic Pneumology, San Camillo Forlanini Hospital, Rome, Italy; ^3^UOC of Anatomopathology, San Camillo Forlanini Hospital, Rome, Italy; ^4^UOSD of Dermatology, San Camillo Forlanini Hospital, Rome, Italy

## Abstract

**Background:**

Severe skin rash is listed among important side effects of EGFR tyrosine kinase inhibitors. Polydatin (PD), a glycosylated polyphenol, is endowed with anti-inflammatory activity in human epidermal keratinocytes.

**Objective:**

This study evaluated the effect of topical application of a moisturizer containing PD to prevent skin rash in patients with mutated non-small cell lung cancer (NSCLC) treated with afatinib.

**Materials and Methods:**

Eligible NSCLC patients with metastatic disease were treated with first-line afatinib 40 mg/die. One day before starting systemic therapy, all patients received topical administration of a 1.5% PD-based cream b.i.d. every day until the end of afatinib treatment.

**Results:**

Out of 34 treated patients, the incidence of skin rash (all grades) was 41.2% and grade 2 rash was 20.6%, and grade 3 rash was not observed in any of the patients. None of the patients discontinued therapy for toxicity. The mean duration of treatment was 6.4 months, calculated from the time treatment was started to the date treatment was stopped.

**Conclusion:**

The results showed that a PD-based cream can reduce the incidence of grade ≥2 skin toxicities in patients treated with afatinib. Clinical study registration number: Prot. No. 130/CE Lazio 1 Italy.

## 1. Introduction

In recent years a substantial progress has been achieved in the treatment of non-small cell lung cancer (NSCLC) through molecular analysis capable of driving the development of more efficient and selective targeted therapy [1].

The epidermal growth factor receptor (EGFR or ErbB1 or HER1), a tyrosine kinase receptor, can activate a wide range of signalling pathways leading to cell growth, proliferation, and survival [2]. Overexpression of EGFR is strongly associated with the development and progression of several malignant tumours, including advanced NSCLC [3]. EGFR is overexpressed and frequently mutated in up to 40–80% of NSCLC and has been considered a good candidate as therapeutic target. The two most common mutations are exon 19 deletions (60%) and L858R missense substitutions at position 858 (35%), where leucine is replaced by arginine, leading to constitutive activation of the receptor [4, 5]. Mutant EGFR can be inhibited either by low-molecular-weight tyrosine kinase inhibitors (TKIs such as gefitinib, afatinib, and erlotinib) or monoclonal antibodies (e.g. cetuximab) [4–7]. Afatinib is a potent second-generation irreversible ErbB family blocker that inhibits tyrosine kinase activity of EGFR and all relevant ErbB family dimmers [8]. In recent clinical trials, afatinib alone was found to be superior to platinum-based doublet chemotherapy in terms of either progression-free survival or overall survival of non-pretreated NSCLC patients with activating EGFR mutations [9–12].

In general, the cutaneous toxicities associated with these targeted agents can potentially affect patient quality of life and treatment compliance and predispose the skin to bacterial, fungal, or viral infections. It is urgently needed to adopt therapeutic and preventive strategies for the management of such toxicities to continue the treatment, maintaining maximal patient tolerability and avoiding treatment delays and interruptions [13]. Strategies to reduce EGFR-TKIs-related adverse events are expected to obtain superior clinical outcomes, a better compliance, and an improved quality of life for patients with advanced NSCLC [14]. Considering the severe local skin toxicity, the treatment is based on drugs capable of reducing mainly the inflammatory cell recruitment. Polydatin (PD, 3,4′,5-trihydroxystilbene-3-*β*-mono-d-glucoside, also known as piceid) is a polyphenol extracted from the root stem of a traditional Chinese herb named *Polygonum cuspidatum* [15]. Among a number of different pharmacodynamic properties, PD has shown potent anti-inflammatory [16–19], antioxidant [20, 21], antiallergy [22], and anticancer activities [23]. Furthermore, polyphenols as PD can interfere in the EGFR system in human keratinocytes, and this effect may be implicated in the regulation of inflammatory and repair-related processes in the skin [24, 25]. In addition, PD induces *β*-defensin production reducing inflammatory response [26], and preliminary human *in vivo* studies showed that daily dietary administration of PD significantly reduced lipid peroxidation levels [27].

All these data prompted us to consider cutaneous application of PD as protective treatment in afatinib-induced skin rash. The present retrospective pilot study evaluated the protective effect of topical application of a cream preparation containing PD against afatinib-induced skin rash in patients with EGFR-mutated stage IV NSCLC.

## 2. Materials and Methods

### 2.1. Patient Selection

Adult patients (age ≥ 18 years) with a histologic or cytologic documented diagnosis of metastatic stage IV NSCLC harbouring activating EGFR common mutations were considered. However, only patients with an Eastern Cooperative Oncology Group (ECOG) performance status of 0 to 2, capable of receiving first-line afatinib 40 mg/die treatment, were eligible for the study. Main exclusion criteria were poor patient compliance, allergic/sensitive to PD, ongoing or previous treatment with other antioxidant topic or oral drugs, and concomitant skin diseases.

### 2.2. Study Design and Treatments

The trial was specifically designed to evaluate a topical protective treatment of EGFR-mediated skin toxicity in order to minimize dose reduction or treatment discontinuation. Patients, after a primary dermatologic visit, received a daily skin treatment, staring from 24 hours before their first dose of afatinib for the duration of antitumor therapy, for at least 3 months. Skin proactive treatment included SPF 30 UVA/UVB nonocclusive sunscreen and a 1.5% PD-based cream (GHIMAS) applied twice a day on the face and the whole body (including the periungual zone). The skin was cleansed with water-emulsified vegetable oils to ensure “affinity cleaning” which would avoid the depletion of the hydrolipid film, according to EPO recommendation [28]. The patients were monitored every 7 days for the first month and after every 20 days or as needed.

The scale used for grading skin toxicities induced by EGFR-targeted therapies was the National Cancer Institute Common Terminology Criteria for Adverse Events (NCI-CTCAE) grading scale, version 3.0 (see Table 1).

The study was approved by the ethics committee at the participating centre and was conducted in accordance with the Declaration of Helsinki (version 2000) and Good Clinical Practice guidelines.

Data on the severity, duration, and management of TKI-induced skin toxicity were analysed in all the treated patients by means of a descriptive statistics.

## 3. Results and Discussion

Thirty-four patients treated in the Oncologic Pneumology Unit of the Azienda Ospedaliera San Camillo-Forlanini, Rome, were suitable for the analysis.  Table 2 shows the main characteristics of the patients. In particular, 75% of patients were aged over 65 years and 91% were nonsmokers. The median follow-up period was 6 months. The mean duration of treatment was 6.4 months, calculated from the time treatment was started to the date treatment was stopped.

The results are illustrated in Table 3. The incidence of skin rash (all grades) was 41.2% and grade 2 rash was 20.6%, and grade 3 rash was not observed in any of the patients. None of the patients discontinued therapy for toxicity. The rash includes tenderness, papulo-pustules, and periungual inflammation. The rash is characterized by interfollicular- and follicular-based erythematous papules and pustules, without microcomedones and comedones characteristic of acne, and is usually observed during the first 2 weeks of therapy.

The management of TKI-induced skin toxicities should be considered as a prerequisite for maintaining patient quality of life in the course of EGFR-targeted therapy [28].

A review on the incidence and severity of rash and other dermatologic adverse effects in selected phase II and III trials reported by the literature show that 45%–100% of patients develop rash [29]. The cutaneous eruptions appear primarily on the face, neck, and upper trunk; the face is often the first area affected by the rash. The rash tends to wax and wane during therapy, with “flare ups” occasionally noted following infusions. Skin lesions resolve without scarring after the withdrawn of the treatment [30].

The pathophysiology of EGFRI-associated skin rash is not completely understood. It is reasonable to assume that anti-EGFR therapy could interfere with the proliferation, differentiation, migration, and attachment of keratinocytes. Moreover, as already mentioned, treatment with TKI could be able to recruit inflammatory cells adversely affecting cutaneous tissues. Since EGFR is highly expressed on epidermal keratinocytes, sebaceous glands, and epithelium of the hair follicle, the inhibition of these receptors can produce characteristic negative dermatologic effects [31].

A National Comprehensive Cancer Network (NCCN) consensus panel, based on expert opinion, reported treatment recommendations for the skin rash. The patients should use emollients for the dry skin, or xerosis, that accompanies anti-EGFR therapies. Moreover, a sunscreen cream application is mandatory, and indeed, the inhibition of EGF receptors produces the loss of the protective function against detrimental effects of sun exposure resulting in worse rash symptoms. Moreover, sunscreen products should have a high sun protection factor. On the other hand, moderate rash symptoms may not require intervention, and, if needed, clindamycin gel and/or topical hydrocortisone may be adequate. It should be pointed out that limited rash symptoms can also be managed with the combination of pimecrolimus 1% plus a tetracycline analogue agent, such as oral doxycycline or minocycline (100 mg twice daily). In contrast, severe rash requires dose interruption of anti-EGFR agents, tetracycline analogue treatment and application of hydrocortisone cream, clindamycin gel, or pimecrolimus, plus oral administration of anti-inflammatory corticosteroids [32].

In addition, PD can interfere in the EGFR system in human keratinocytes, and this effect may be involved in the regulation of inflammation and repair-related processes in the skin [25–27, 33–35]. Anti-EGFR treatment typically gives a rash with histological features of a typical dermatoses induced by keratinocyte-interleukin-8 production. Since PD is able to modulate interleukin-8 gene expression, it is possible to hypothesize that PD could act, at least in part, through an interleukin-8 inhibition mechanism [17, 20, 27].

In our study, the incidence of rash (all grades) was 41.2% (20.6% of grade 2), without any event of grade 3 and no withdrawal of treatment for skin adverse events. This incidence of rash is lower compared to that reported in literature; actually, in clinical trials with first-line afatinib, the overall incidence of rash was between 60% and 80%. Two of the largest trials are LUX-Lung 3 and LUX-Lung 6 [36, 37]. These trials have a comparable design with the exception of the platinum-based chemotherapy regimen: pemetrexed/cisplatin in LUX-Lung 3 and gemcitabine/cisplatin in LUX-Lung 6. In these studies, the patients, whose tumours have common EGFR mutations, receiving first-line afatinib showed a progression-free survival. In these patients, the most common adverse events (grade 3 and 4) related to afatinib in comparison with chemotherapy were rash/acne, diarrhoea, paronychia, and stomatitis/mucositis [36, 37]. In the LUX-Lung 3 trial, the treatment was discontinued because of treatment-related adverse events in 8% of patients. The incidence of rash (all grades) was 89.1%, grade 1-2 rash was 72.9%, and grade 3 rash was 16.2%. In the LUX-Lung 6, the adverse events-related drug withdrawal rate was lower than in LUX-Lung 3 trial (2.1%), as well as the incidences of rash (all grades: 80.8%: grade 1-2: 66.1%; grade 3: 14.2%). This could be explained by the fact that the treating physicians became more confident in the management of the side effects of afatinib and what was earlier perceived as higher grade toxicity was now thought to be of lower severity. In addition, Kudo et al. [38] retrospectively evaluated 49 consecutive patients with EGFR-mutant NSCLC treated with afatinib between 2009 and 2015. The results showed that grade ≥2 skin rash occurred in 17 patients (35%), 5 of them (10%) during the first week after the initiation of afatinib therapy. Compared to this report, our results look better because our global incidence of skin rash, including grade 1 events, is 40.2% versus 35% of grade ≥2 events. Another open-label, randomized, controlled trial evaluated the prophylactic effect of tetracycline versus no treatment in reducing afatinib skin rash in 90 NSCLC patients receiving afatinib 40 mg/day. Rash incidence of any grade and grade ≥2 was lower in the tetracycline arm with respect to the control arm (44.5 vs. 75.6%, *p*=0.046, and 15.6 vs. 35.6%, *p*=0.030, respectively). Compared to this report [39] we can observe that in our results the protective effect of PD is equivalent to that of a prophylaxis with tetracyclines, a well-recognized therapeutical strategy for the prevention of anti-EGFR skin rashes.

The present study, limited in the number of patients and very preliminary, suggests that a PD-based cream can reduce the incidence of grade 2-3 or greater skin toxicities without additional side effects in patients treated with afatinib. The results are compared with the toxicity data of LUX-Lung 3 and 6 trials. Topical treatments in alternative to oral antibiotics deserve to be considered, as topical treatment approaches are used extensively in this setting. Additional well-controlled prospective clinical trials are needed to further examine the potential benefits of prophylactic skin-treatment of EGFR-mediated toxicities and to establish a framework for consistent evidence-based treatment approaches based on biological mechanisms.

## 4. Conclusion

In this preliminary study, the activity of polydatin (PD), a well-tolerated natural extract, has been evaluated for the prophylaxis of skin toxicity during an afatinib-based treatment, avoiding the use of antibiotics.

These results suggest that a proactive prophylactic management of skin rash with a PD-based cream can reduce the incidence of grade ≥2 skin toxicities without additional side effects in patients treated with afatinib.

## Figures and Tables

**Table 1:** Skin toxicity grading according to the National Cancer Institute Common Toxicity Criteria for Adverse Events (NCI-CTCAE).

Grade	Description
1	Papules and/or pustules covering <10% BSA, which may or may not be associated with symptoms of pruritus or tenderness
2	Papules and/or pustules covering 10–30% BSA, which may or may not be associated with symptoms of pruritus or tenderness; associated with psychosocial impact; limiting instrumental ADL
3	Papules and/or pustules covering >30% BSA, which may or may not be associated with symptoms of pruritus or tenderness; limiting self-care ADL; associated with local superinfection with oral antibiotics indicated
4	Papules and/or pustules covering any % BSA, which may or may not be associated with symptoms of pruritus or tenderness and are associated with extensive superinfection with IV antibiotics indicated; life-threatening consequences
5	Death

BSA: body surface area; ADL: activities of daily living.

**Table 2:** Patient characteristics (*n*=34).

	Number of patients	Percentage (%)
*Age*		
<65	6	25
>65	28	75
*Gender*		
Female	18	53
Male	16	47
*Mutation*		
Exon 19	22	65
Exon 21	12	35
*ECOG*		
0-1	30	88
2	4	12
*Smoking status*		
Smoker	3	9
Nonsmoker	31	91

**Table 3:** Skin-rash incidence.

	All grades (*n*, %)	Grade 1 (*n*, %)	Grade 2 (*n*, %)	Grade 3 (*n*, %)
Skin rash	14 (41.2)	7 (20.6)	7 (20.6)	0
Female	8 (23.5)	2 (5.9)	3 (8.6)	0
Male	6 (17.6)	5 (14.7)	4 (12)	0

## Data Availability

The data used to support the findings of this study are available from the corresponding author upon request.

## Abbreviations

ADL: Activities of daily living
ALT: Alanine aminotransferase
AST: Aspartate aminotransferase
BSA: Body surface area
CTCAE: Common Terminology Criteria for Adverse Events
ECOG: Eastern Cooperative Oncology Group
EGFR: Epidermal growth factor receptor
HER2: Human epidermal growth factor 2
NCCN: National Comprehensive Cancer Network
NSCLC: Non-small cell lung cancer
OS: Overall survival
PFS: Progression-free survival
TKIs: Tyrosine kinase inhibitors.
